# Improving Large Language Models’ Summarization Accuracy by Adding Highlights to Discharge Notes: Comparative Evaluation

**DOI:** 10.2196/66476

**Published:** 2025-07-24

**Authors:** Mahshad Koohi Habibi Dehkordi, Yehoshua Perl, Fadi P Deek, Zhe He, Vipina K Keloth, Hao Liu, Gai Elhanan, Andrew J Einstein

**Affiliations:** 1 Department of Computer Science New Jersey Institute of Technology Newark, NJ United States; 2 Department of Informatics New Jersey Institute of Technology Newark, NJ United States; 3 School of Information Florida State University Tallahassee, FL United States; 4 Department of Medical Informatics Yale University New Haven, CT United States; 5 Department of Computer Science Montclair State University Montclair, NJ United States; 6 School of Medicine University of Nevada Reno, NV United States; 7 Department of Medicine Columbia University Irving Medical Center New York, NY United States

**Keywords:** electronic health record, EHR, EHR summaries, clinical notes summarization, discharge notes summarization, LLM summaries, ChatGPT summaries, highlighted EHR notes, accuracy of summaries, discharge notes, large language model, LLM, ChatGPT, artificial intelligence, AI

## Abstract

**Background:**

The American Medical Association recommends that electronic health record (EHR) notes, often dense and written in nuanced language, be made readable for patients and laypeople, a practice we refer to as the simplification of discharge notes. Our approach to achieving the simplification of discharge notes involves a process of incremental simplification steps to achieve the ideal note. In this paper, we present the first step of this process. Large language models (LLMs) have demonstrated considerable success in text summarization. Such LLM summaries represent the content of EHR notes in an easier-to-read language. However, LLM summaries can also introduce inaccuracies.

**Objective:**

This study aims to test the hypothesis that summaries generated by LLMs from highlighted discharge notes will achieve increased accuracy compared to those generated from the original notes. For this purpose, we aim to prove a hypothesis that summaries generated by LLMs of discharge notes in which detailed information is highlighted are likely to be more accurate than summaries of the original notes.

**Methods:**

To test our hypothesis, we randomly sampled 15 discharge notes from the MIMIC III database and highlighted their detailed information using an interface terminology we previously developed with machine learning. This interface terminology was curated to encompass detailed information from the discharge notes. The highlighted discharge notes distinguished detailed information, specifically the concepts present in the aforementioned interface terminology, by applying a blue background. To calibrate the LLMs’ summaries for our simplification goal, we chose GPT-4o and used prompt engineering to ensure high-quality prompts and address issues of output inconsistency and prompt sensitivity. We provided both highlighted and unhighlighted versions of each EHR note along with their corresponding prompts to GPT-4o. Each generated summary was manually evaluated to assess its quality using the following evaluation metrics: completeness, correctness, and structural integrity.

**Results:**

We used the study sample of 15 discharge notes. On average, summaries from highlighted notes (H-summaries) achieved 96% completeness, 8% higher than the summaries from unhighlighted notes (U-summaries). H-summaries had higher completeness in 13 notes, and U-summaries had higher or equal completeness in 2 notes, resulting in *P*=.01, which implied statistical significance. Moreover, H-summaries demonstrated better correctness than U-summaries, with fewer instances of erroneous information (2 vs 3 errors, respectively). The number of improper headers was smaller for H-summaries for 11 notes and U-summaries for 4 notes (*P*=.03; implying statistical significance). Moreover, we identified 8 instances of misplaced information in the U-summaries and only 2 in the H-summaries. We showed that our findings supported the hypothesis that summarizing highlighted discharge notes improves the accuracy of the summaries.

**Conclusions:**

Feeding LLMs with highlighted discharge notes, combined with prompt engineering, results in higher-quality summaries in terms of correctness, completeness, and structural integrity compared to unhighlighted discharge notes.

## Introduction

### Background

Electronic health records (EHRs) [1] are digital versions of a patient’s medical records that were originally intended for communication among health care professionals, such as clinicians and nurses, to facilitate quick comprehension and efficient interoperability. These notes are typically written in highly technical language, filled with abbreviations, complex sentence structures, and medical jargon that may be unfamiliar to individuals without a medical background [2].

According to the 21st Century Cures Act and the Open Notes Rule [3], it is required to make these notes available to patients through patient portals, and because of this, a significant challenge has emerged. Patients with limited health literacy may struggle to understand written medical information, communicate health concerns with health care providers, and navigate EHR systems [2]. Research shows that patients often use different vocabulary than clinicians when searching for health information [4], indicating that the language clinicians use in discharge notes is potentially unfamiliar and difficult for patients to understand. To increase the comprehension of EHR notes, the American Medical Association recommends a grade 6 reading level, whereas the National Institutes of Health (NIH) recommends a grade 7 to 8 reading level for EHR notes [5,6]. The National Cancer Institute recommends an grade 6 reading level, reflecting the average reading level for a US citizen [7-9].

Large language models (LLMs) [10-13] have demonstrated success in text summarization and simplification tasks [14-16]. ChatGPT (OpenAI) [17], in particular, has shown promise in generating summaries that are comparable to those created by human experts [18]. Prompt engineering [19,20] has gained significant popularity in recent years due to its potential to improve the performance of LLMs. By carefully crafting prompts, we can instruct LLMs on how to extract key information, maintain coherence, and structure summaries according to specific needs. Well-designed prompts help control the level of detail, tone, and style, thereby improving both accuracy and readability [19,21]. Because LLMs rely on input instructions [22], prompt engineering is essential for aligning model behavior with the desired summarization outcome, reducing ambiguity and enhancing reliability.

Despite the advantages, several concerns remain regarding the use of LLMs in the medical field, particularly for summarizing medical texts [23,24]. These include issues of reliability, inconsistency in output quality, the potential for false information, and prompt sensitivity. Without addressing these concerns, LLMs might not realize their full potential for summarizing discharge notes. As mentioned earlier, the presence of inadequate sentence structure, a lack of punctuation, the presence of fragmented expressions, and an abundance of abbreviations within discharge notes make summarization relatively more difficult compared to general domain text summarization. Therefore, LLM-generated summaries may sometimes omit crucial information or lack proper structure.

In this paper, we present the first step in the process of simplifying discharge notes by harnessing the summarization capabilities of LLMs. In this study, we aim to generate more accurate, structured summaries from discharge notes where headers provide clear orientation and make the content easier to understand [25]. These summaries are then converted into accurate, simplified notes that are understandable for patients with a grade 6 reading level [26].

To address and evaluate the loss of crucial information in the summaries of discharge notes, our approach involves modifying the input by providing LLMs with highlighted discharge notes, where detailed information is emphasized. We then compare these summaries with those generated from unhighlighted notes. Our previous research [27,28] developed an innovative method for automatically highlighting detailed information in discharge notes. In this study, we automatically highlighted discharge notes using that technique (which is described in the Methods section). Our technique also uses prompt engineering to enhance the structural integrity of the generated summaries and address prompt sensitivity. We hypothesize that feeding LLMs with the highlighted notes aids in summarizing them more accurately. The rationale for this prediction is that, with a prompt to focus on the highlighted detailed information in a text, the summary will be more accurate and better structured than one obtained without highlighting.

We conducted this study to test the following hypothesis: Summaries generated by LLMs from highlighted discharge notes will achieve increased accuracy compared to those generated from the original notes. This is done by generating summaries from both unhighlighted and highlighted discharge notes using LLMs and then comparing both versions of the summaries for each discharge note to see which method yields better results. We evaluated different dimensions of the accuracy of summaries with proper metrics, including completeness, correctness, and structural integrity. Our findings support the hypothesis that summarizing highlighted discharge notes improves the accuracy of the summaries as measured by these metrics. Thus, it is advantageous to generate summaries of discharge notes after highlighting them. Structured summaries of highlighted discharge notes provide a valuable starting point for future efforts to enhance the simplification of these notes while maintaining their accuracy.

### Related Work

The goal of text summarization is to condense text while keeping its key information and important content intact [29,30]. Before the advent of LLMs, text summarization was primarily performed by automatic text summarization (ATS) [31-33]. ATS involves a trainable summarizer that considers various features, such as sentence position, keywords, sentence centrality, resemblance to the title, inclusion of named entities and numerical data, relative length, bushy path, and aggregated similarity to generate summaries [34].

While traditional ATS methods are still in use, LLMs are increasingly adopted for summarization tasks as they have shown great performance in this area. Therefore, there has been a significant shift from ATS to LLMs, with methods evolving from pretraining and fine-tuning to prompt-based approaches [31]. Besides, when summarizing discharge notes, mainly replete with medical abbreviations, LLMs typically spell out most of the abbreviations, yielding more readable clinical notes.

In a recent study [35], 8 LLMs (Flan-T5, Flan-UL2, Alpaca, MedAlpaca, Vicuna, Llama-2, GPT-3.5, and GPT-4) were evaluated for various clinical text summarization tasks. The study identified GPT-4 as the best-performing model, particularly when using in-context learning [36] for adaptation. GPT-4 demonstrated superior completeness, correctness, and conciseness in summaries compared to other models and even human experts. Therefore, in this study, we have used the latest version of ChatGPT, GPT-4o, for summarization.

In the study by Kanwal and Rizzo [37], they developed an extractive summarization method tailored for discharge notes using a bidirectional encoder representations from transformers–based model fine-tuned on the MIMIC-III dataset. Their approach uses attention scores from the final transformer layer to identify clinically important sentences without relying on reference summaries. The model dynamically selects sentences with above-average attention scores, aiming to preserve essential clinical content. Their method outperformed 3 baseline approaches, namely frequency-based, graph-based, and K-means centroid-based extractive summarization, demonstrating better content preservation and semantic alignment with the original notes.

In the study by Alsentzer and Kim [38], they explored extractive summarization of discharge notes from the MIMIC-III database. They estimated an upper bound on extractive summarization by measuring how much of the information in a discharge summary can be found elsewhere in the patient’s EHR notes using concept unique identifiers. To support future summarization tasks, they also developed a long short-term memory model to label word-level topics in the “history of present illness” sections. This classifier achieved an *F*_1_-score of 0.876 on a manually annotated test set, demonstrating its potential for generating topic-specific evaluation datasets for extractive summarization models.

In the study by Ma et al [39], the researchers developed a method called ImpressionGPT to summarize the “impression” section of radiology reports using ChatGPT. They used a dynamic prompt generation and iterative optimization approach to improve the performance of ChatGPT in this task. The results showed that ImpressionGPT achieved better performance in generating correct and concise summaries compared to existing methods, demonstrating its potential to enhance clinical workflows and reduce the workload of health care professionals.

The challenges faced by clinicians with limited time to remain abreast of the rapidly expanding medical literature have been documented in the study by Hake et al [40]. The authors evaluated the performance of ChatGPT in summarizing 140 peer-reviewed medical abstracts from 14 different journals. Their methodology involved prompting ChatGPT to create summaries and then having physicians assess the quality, accuracy, and bias of these summaries, where bias refers to the phenomenon where artificial intelligence (AI) systems are trained on data that lack sufficient reflection of the diversity within the population. The results showed that ChatGPT-produced summaries were 70% shorter than the original abstracts but maintained an accuracy of 92.5%. The study concluded that while ChatGPT can aid in summarizing medical literature, full-text evaluation remains crucial for critical medical decisions. Although they conducted the study on scientific paper abstracts, which are well structured and have much simpler language than discharge notes, they reported several occurrences of serious inaccuracies in ChatGPT summaries that could materially impact the major interpretation of the text.

## Methods

### Overview

Existing research on summarization using ChatGPT [35] has primarily focused on prompt engineering to achieve optimal results. These studies have not explored the potential benefits of modifying the input format and providing customized context tailored to the specific text. In contrast, our study goes beyond prompt engineering by incorporating important information directly into the input text. This approach ensures that ChatGPT pays attention to key details, enhancing the completeness, correctness, and structural integrity of the generated summaries.

In the Methods section, we first explain the automatic EHR highlighting technique, followed by the summarization method used in this study.

### Automatic EHR Highlighting

In our previous studies [27,28], we developed a cardiology interface terminology (CIT) to facilitate efficient highlighting of detailed content in cardiology-related discharge notes. The process is composed of 2 phases. In the first phase [27], we created an initial version of CIT (ICIT), which contains the cardiology-related subhierarchies of Systematized Nomenclature of Medicine–Clinical Terms [41,42]. However, ICIT did not capture all the important information from the discharge notes of cardiology patients. Therefore, we added concepts by extracting fine-granularity phrases from discharge notes that contained ICIT concepts. This was done using a semiautomatic iterative process, resulting in the formation of CIT. In each iteration, we highlighted the build dataset with the last version of the CIT and calculated the coverage and breadth of the highlighted dataset. Coverage is the percentage of the total number of words that are highlighted in one note, and breadth is the average length of each highlighted concept.

We defined 2 operations for mining more complex, higher-granularity phrases from the build dataset: concatenation and anchoring. Concatenation combined adjacent highlighted concepts, potentially separated by stop words, to form a meaningful phrase. Anchoring expanded existing highlighted concepts by attaching surrounding words on the left, right, or both sides. We applied an iterative process in which the application of the concatenation operation was followed by the application of the anchoring operation.

Because CIT is an interface terminology, its concepts should follow the requirements for being considered concepts of a terminology. According to the desiderata of Cimino et al [43], a concept is an embodiment of a particular meaning. Concept orientation means that terms must correspond to at least one meaning (“nonvagueness”) and no more than one meaning (“nonambiguity”) and that meanings correspond to no more than one term (“nonredundancy”) [43,44].

After each application of either concatenation or anchoring, the newly mined phrases, as potential CIT concepts, were manually and automatically reviewed. Phrases that were deemed legitimate during the review, both structurally and semantically, such as “normal ejection fraction,” were accepted and added to the CIT. All illegitimate phrases, whether identified automatically or manually, were added to a rejection list. For example, “step down unit for further” was rejected because it is a truncated phrase with an incomplete meaning. After each application of either the concatenation or anchoring operation, we highlighted the build dataset with the updated version of the CIT. This iterative process continued until the increase in coverage became negligible (<2% increase). The results of this phase served as training data for the second phase, where all the concepts in the resulting CIT were considered positive samples (labeled 1), and all the illegitimate phrases in the rejection list were considered negative samples (labeled 0). More detailed descriptions, including examples, are given in the study by Dehkordi et al [27].

**Figure 1:**
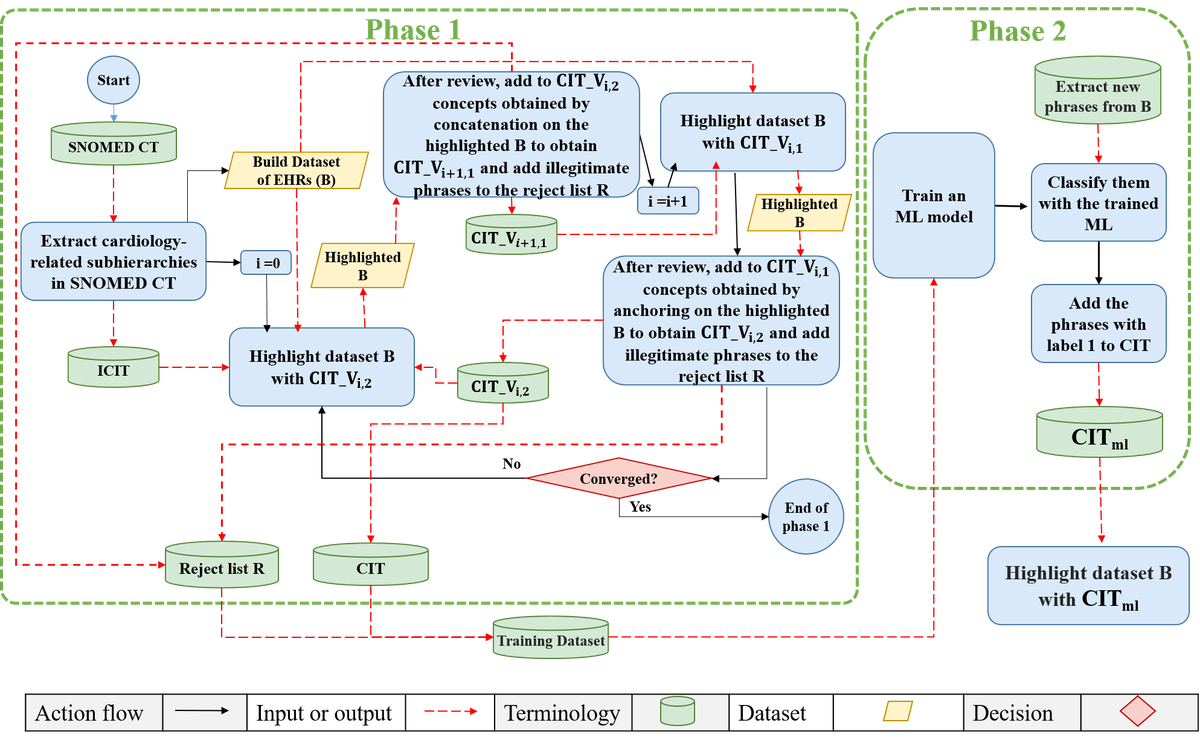
Diagram of constructing CITml. Cardiology interface terminology (CIT) has versions with 2 indices. The first indicates the iteration number, and the second is binary, with 1 following concatenation and 2 following anchoring. CIT_V: updated version of the CIT; EHR: electronic health record; ICIT: initial version of the CIT; ML: machine learning; SNOMED CT: Systematized Nomenclature of Medicine–Clinical Terms.

In the second phase [28], we first trained a feedforward neural network model using the training data obtained from the first phase. Next, we extracted more phrases from discharge notes and applied predefined rules to filter out structurally illegitimate ones. The remaining phrases were labeled using the neural network model. The accepted phrases were then added to the CIT, forming CIT_ml_. CIT_ml_ significantly improved the evaluation metrics of highlighted discharge notes. More detailed descriptions, including examples, are given in the study by Dehkordi et al [28]. Figure 1 shows the diagram of constructing CIT_ml_.

In another study [45], we created and assessed interface terminologies designed to extract pertinent details from the clinical records of patients with COVID-19, underscoring the generalizability of our methodologies.

### Data

We randomly selected 15 discharge notes from the MIMIC-III database from 2 intensive care units related to cardiology patients: the coronary care unit and the cardiac surgery recovery unit. Only the “discharge summary” category was extracted from the discharge notes. We chose 15 notes to enable a careful manual comparison of 2 summaries per note, with and without highlighting, versus the original text.

For each note, we created 2 HTML files: one containing the original discharge note without highlights and the other containing the highlighted discharge note, which is the output of the highlighting technique from our previous work.

As described in the Methods section, our previous work involved curating an interface terminology leveraging machine learning techniques. This terminology encompasses fine-granular, detailed concepts found in discharge notes. In this study, we applied this curated interface terminology to highlight the 15 discharge notes, such that all concepts from the terminology that were present in these notes were highlighted. The highlighted information was marked using span tags with a blue background color with the color code of #ADD8E6. Moreover, we created plain text in HTML format to ensure both inputs have a uniform format to be provided to ChatGPT. The plain text HTML file was created by enclosing the entire text within a <p></p> tag.

To provide insights into the 15 discharge notes we were summarizing, Table 1 presents the text volume, overall themes, word frequency, and the repetition count of each word within a single document.

**Table 1:** Text volume, overall themes, and word frequency of 15 discharge notes.

Theme	TW^a^	SW^b^	TW_ESW^c^	DW_ESW^d^	N1^e^	N2^f^	N3^g^
Management of shortness of breath and heart failure	164	71	93	83	74	8	1
Aortic valve replacement	366	122	244	188	152	24	12
Bilateral renal artery stenosis	160	51	109	77	58	12	7
Three-vessel coronary artery bypass grafting	266	103	163	122	96	19	7
Aortic valve replacement and coronary artery bypass grafting	377	93	284	203	169	16	18
Peripheral vascular disease	448	153	295	206	156	31	19
Septic shock with respiratory failure	386	113	273	220	187	20	13
Coronary artery disease	312	101	211	144	106	27	11
Diabetic ketoacidosis with a history of coronary artery disease	180	41	139	112	97	12	3
Posterior mediastinal mass	560	199	361	255	197	36	22
Cardiac arrest	520	211	309	251	205	39	7
Hypercalcemia	375	133	242	198	165	26	7
Right coronary artery disease	411	160	251	168	119	33	16
Coronary artery bypass grafting	155	61	94	80	70	9	1
Aortic valve replacement	128	46	82	76	70	6	0
—^h^	320 (139)	111 (53)	209 (90)	159 (64)	128 (50)	21 (11)	10 (7)

^a^TW: total words.

^b^SW: stop words.

^c^TW_ESW: total words excluding stop words.

^d^DW_ESW: distinct words excluding stop words.

^e^N1: words repeated once, excluding stop words.

^f^N2: words repeated twice, excluding stop words.

^g^N3: words repeated more than 2 times, excluding stop words.

^h^Not applicable.

### Prompt Engineering

Prompt engineering [19,20,46] is used to craft and refine input prompts to achieve the best possible results from LLMs, such as ChatGPT. However, it is a challenging task because even a minor change can significantly impact performance, leading to different results [39].

We proposed a protocol to incrementally generate effective prompts for both types of summaries from highlighted and unhighlighted notes.

In prompt engineering, we need to use static input and simply refine the prompt to find an appropriate prompt, which leads us to the best possible result. Therefore, we chose a random note that was not used in the evaluations to develop and test the prompt engineering design. We needed 2 prompts because 2 summaries are required for each note, the summary from unhighlighted notes (U-summary) and the summary from highlighted notes (H-summary).

We began the prompt engineering process with the unhighlighted text. To generate summaries, we initially used a simple prompt, “Give me a summary of this note.” We evaluated the completeness, correctness, word count, and structure of the generated summaries. If the summaries were too long, erroneous, or incomplete, we adjusted the prompt accordingly. For instance, the first prompt, “Give me a summary of this note,” resulted in a summary longer than the original text, so we revised it to “Give me a short summary of this note.” In another example, we determined that the output was not structured, so we added “structured” to the prompt, forming the prompt “Give me a short structured summary of this note,” so the summary would include headers and bullets for better orientation. This iterative process continued until the results were satisfactory to us after measuring the relevant metrics. Once we finalized the prompt for the unhighlighted text, we adapted it for the highlighted text by adding information about the format of the highlighted information.

Finally, to ensure that the prompts were generalizable to other clinical notes, we applied the final prompts to 3 more randomly selected notes that were not used in the evaluation of this study. Because we obtained satisfactory summaries, after measuring the relevant metrics for both U-summaries and H-summaries, we concluded that the final prompts were suitable for our study.

The final prompts were as follows:

- The prompt for the unhighlighted note was “Give me a short structured summary of this EHR note”
- The prompt for the highlighted note was “Give me a short structured summary of this EHR note, focusing on the highlighted information [whose tags are “style=”‘background-color: #ADD8E6’]”

### Generating Summaries

For each of the 15 randomly selected notes, we provided both the prompts to GPT-4o, along with the corresponding HTML files. To measure the evaluation metrics, we extracted all important information items from the original notes. Consequently, we manually inspected both summaries for each such item. For each summary, we counted the number of words, the number of important information items included by comparing them to the list of all important information items, and the number of possible erroneous statements generated by ChatGPT. We also counted the structural elements, misplaced information items, and false information items. We used this information to calculate the evaluation metrics.

### Evaluation Metrics

#### Overview

Assessing the quality of a summary is challenging, and currently, no automatic proxy exists for this task. Therefore, similar to related studies [35], extensive manual effort is required to measure various aspects and compare which summary is more accurate. The evaluation was done by MKHD, a third-year PhD candidate in computer science with 3 years of research experience in processing EHR notes who has published studies [26-28,47,48] on this subject under the supervision of YP, who has more than 30 years of experience in medical informatics research. We used existing metrics and proposed new metrics for evaluating several aspects of the structured summaries generated. Our purpose was to test the following hypothesis: The H-summaries of discharge notes are likely to be more accurate than the U-summaries of those notes.

To assess the plausibility of the hypothesis, we needed to consider several metrics. The following 4 aspects should be evaluated in a summary: completeness, correctness, succinctness, and structural integrity. First, the completeness and correctness of the summary must be assessed. We followed the study by Van Veen et al [35] in using the metrics for completeness and correctness, which, respectively, measured the extent to which important information was covered and how accurate the covered information was. Second, the structure of the summary should be assessed by examining all the headers to identify proper and improper headers as well as bullets, which contribute to secondary structuring. In addition, because correct information might be placed under inappropriate headers within the text, we proposed a metric called the misplaced information to evaluate this aspect of each summary. Furthermore, the succinctness of the summary could be evaluated by the percentage reduction in size compared to the original text, which we referred to as the length reduction metric. This metric, although interesting, was not related to the quality of the summary.

#### Completeness

The completeness metric measures how well the summary has captured the important information from the original text. It is calculated as the percentage of the important information items included in the generated summary compared to all the important information items in the original text. This metric has also been used in other studies [35,49,50] to evaluate summaries based on the original text. Completeness can be calculated using the following equation:

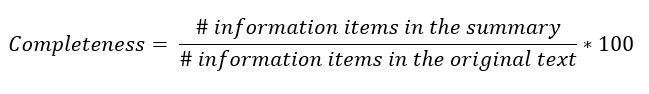



**Figure 2:**
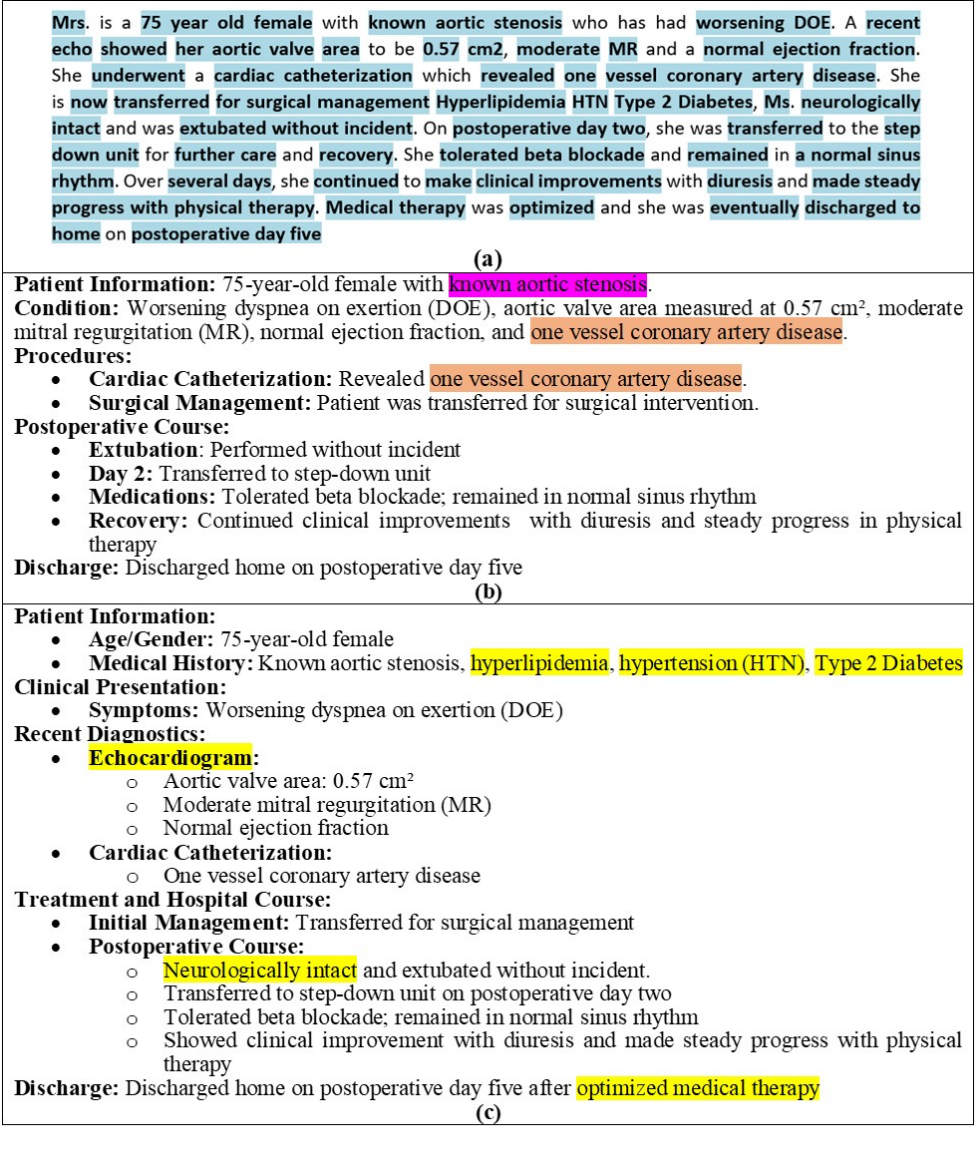
(A) An original highlighted note with 128 words, (B) the summary from unhighlighted notes of the note with 91 words and 84% (31/37 items of information) completeness, (C) and the summary from highlighted notes of the same note with 111 words and 100% completeness. The pink highlight in (B) indicates misplaced information. The orange highlight in (B) indicates repetitive information, the first of which is redundant and misplaced. The yellow highlights in (C) indicate information items from the original text that do not appear in (B).

Examples of important information items in Figure 2A are “known aortic stenosis,” “worsening DOE,” and “extubated without incident.”

To calculate completeness, we first extracted and listed all information items from the original notes. We examined each summary, identifying how many of these items were present in the summary. We calculated the completeness of each note using equation 1. We calculated the average completeness scores for the 15 H-summaries and the 15 U-summaries separately.

#### Correctness

A summary is considered correct if it does not contain erroneous information. If it contains at least one instance of inaccurate information, it is then considered incorrect. The correctness of the summaries for a group of notes is the percentage of correct summaries. To evaluate correctness, we manually scanned the 15 U-summaries and the 15 H-summaries to identify all false information items based on the original text. This metric has also been used in similar studies [35,50,51].

#### Structural Elements Count

##### Overview

This metric quantifies the total number of structural elements, including the headers and bullet points, used to organize and structure the summaries. The structural elements count (SEC) is calculated by simply counting all the headers and bullet points in each summary.

ChatGPT added headers and subheaders to both the H-summaries and U-summaries based on the given prompt. Upon manual review of the headers, we found that most of them accurately captured the information that followed, and we referred to these as “proper headers.” However, some headers were found to be redundant or misleading. Redundant headers are unnecessary and do not provide additional orientation for the reader, while misleading headers do not accurately represent the content that follows. Overall, having more proper headers and subheaders enhances the orientation of the note. In contrast, improper headers, whether redundant or misleading, may confuse the reader. Naturally, our goal was to maximize the number of proper headers to improve reader orientation and minimize the number of improper headers to avoid confusion and redundancy.

##### Proper Headers

**Figure 3:**
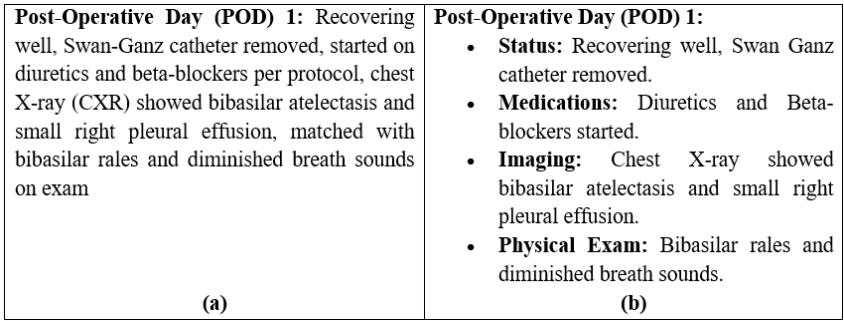
(A) A portion of a summary lacking proper headers and (B) the same portion of the summary with proper headers.

Proper headers are those that correctly and effectively organize the text, accurately capturing the information that follows, leading to a clearer summary. The number of proper headers for each summary is determined by subtracting the improper headers from the total number of headers. For example, Figure 3A shows a portion of a summary where all the information is included in a single block of text, while Figure 3B shows the same portion of the summary with proper headers. In Figure 3B, information regarding status, medications, imaging, and physical examination is correctly categorized under separate sections with appropriate labels, thereby improving the orientation of the note.

##### Improper Headers

**Figure 4:**
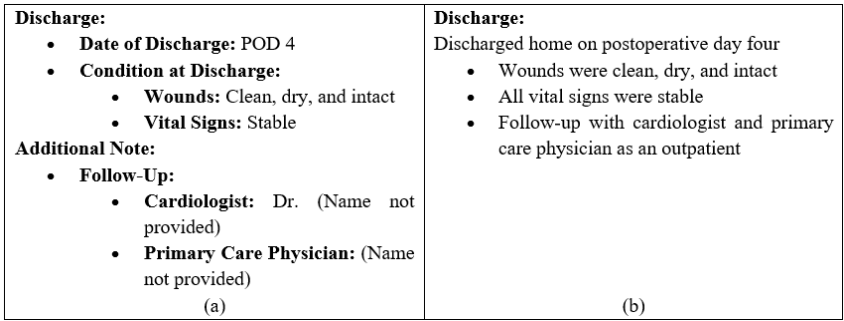
(A) A portion of a summary having improper headers and (B) the same portion of the summary without improper headers.

Figure 4 shows an example of improper headers. Figure 4A shows a portion of a U-summary, and Figure 4B is the corresponding H-summary. As shown, Figure 4A contains numerous redundant subtitles, which significantly interfere with the orientation because they may overwhelm and confuse the reader.

To assess the improper headers metric, we manually reviewed all the summaries to identify the number of additional structural headers in each summary to compare the corresponding summaries with regard to their structural integrity.

##### Misplaced Information

Sometimes, a summary incorrectly categorizes information under the wrong section, resulting in misplaced information, confusing the reader. For example, in Figure 2B, “known aortic stenosis” is placed under the “patient information” category, whereas it should be under a header titled “medical history.” This structure is correctly applied in the highlighted summary shown in Figure 2C. We counted the number of cases of misplaced information in both types of summaries to compare their structural information integrity.

##### Length Reduction

To evaluate the succinctness of the generated summaries, we used the length reduction metric, which is calculated using equation 2 with a Python (Python Software Foundation) program:

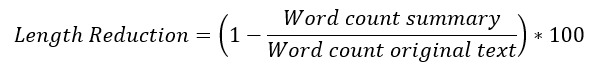



We used the Fisher exact test [52,53] twice; once to compare the completeness metric between the H-summaries and the U-summaries and the second time to compare the number of improper header metrics between the H-summaries and the U-summaries.

### Power Analysis

In our methodology, power analysis [54] was conducted using the Fisher exact test to determine the necessary sample size for detecting significant effects, particularly valuable when dealing with categorical data in scenarios of small sample sizes or sparse data. We started our analysis with the 2 metrics that were dominant for the accuracy of the discharge notes.

#### Completeness Metric Analysis

For assessing the completeness of summaries, we had 2 equal-sized groups, each comprising 15 paired discharge notes. In this evaluation, summaries generated by method 1 (H-summaries) were superior in 13 instances, while method 2 (U-summaries) led in 2 cases. Consequently, the probability of a favorable outcome in group 1 (H-summaries) was 86.7%, and in group 2 (U-summaries), it was 13.3%. Given the consistent performance advantage of H-summaries, a 1-sided Fisher exact test was appropriate, with a significance level (α) set at .025. The resulting power analysis yielded a high power of 0.98, indicating robust confidence in these findings.

#### Improper Header Metric Analysis

For the improper header metric, power analysis using the Fisher exact test was conducted, taking into account unequal sample sizes. The total count of structural elements (SECs), which include headers and bullet points, was 463 for H-summaries, of which 4 (0.9%) were improper, and 516 for U-summaries, of which 30 (5.8%) were improper This metric necessitated a 2-sided test with an α of .05, achieving a perfect power of 1, suggesting that the test reliably confirmed substantially more improper headers for method 2 when compared to method 1.

#### Correctness Metric Analysis

Regarding the correctness metric, all H-summaries contained 870 items of information, 2 (0.2%) of which were false, while the U-summaries contained 795 items, 3 (0.38) of which were false. This resulted in probabilities of 0.2% (2/870) for H-summaries and 0.3% (3/795) for U-summaries. Given the substantial total sample size exceeding 1000 items (1665 items of information in total), a Pearson chi-square test [55] for unequal sample sizes was deemed suitable. However, the resulting power was low at 0.06. Further analysis revealed that to achieve a minimum power of 0.8 with a 2-sided test, the required sample sizes would need to increase to 41,146 items for H-summaries and 37,032 items for U-summaries, which is not practical for manual comparison.

#### Misplaced Information Metric Analysis

Finally, for the misplaced information metric, considering that there are 2 instances of misplaced information in 463 SECs for H-summaries and 8 in 516 SECs for U-summaries, the probabilities are 0.4% (2/463) and 1.6% (8/516), respectively. Conducting a 2-sided Fisher exact test power analysis with α of .05 and considering unequal sample sizes, we found that the resulting power was 0.41, which was low. To achieve a desired power of 0.8, recalculations suggested that sample sizes of 1272 for H-summaries (almost 3 times the current size) and 1145 for U-summaries (almost twice the current size) were needed.

### Ethical Considerations

This study used discharge notes from the MIMIC-III dataset [56], a large, freely available database containing deidentified health-related data from >40,000 patients who were admitted to the Beth Israel Deaconess Medical Center between 2001 and 2012. The database includes information, such as vital signs, laboratory test results, procedures, medications, etc. The MIMIC-III database was approved by the institutional review boards [57] of the Beth Israel Deaconess Medical Center and the Massachusetts Institute of Technology. The requirement for individual patient consent was waived, as the dataset is fully deidentified and does not impact clinical care [58]. Before inclusion in MIMIC-III, all data underwent a rigorous deidentification process in compliance with Health Insurance Portability and Accountability Act (HIPAA) [59] standards [58]. Structured data were deidentified by removing all 18 HIPAA-defined protected health information elements, such as names, addresses, phone numbers, and exact dates [58]. To further ensure privacy, dates were randomly shifted while preserving relative intervals, and patients aged >89 years had their ages masked. Free-text fields, including physician notes and diagnostic reports, were processed using an extensively evaluated deidentification system that used dictionary lookups and pattern-matching algorithms to remove protected health information [58]. Because all data are deidentified and do not contain any identifiable patient information, the requirement for informed consent was waived by the overseeing institutional review boards [58].

## Results

### Overview

Figure 2A shows an example of a discharge note (note 15) with 128 words, accompanied by its U-summary (Figure 2B) and H-summary (Figure 2C), containing 91 and 111 words, respectively. We noted that this discharge note was missing, describing the actual surgery that obviously happened during hospitalization of the patient. We assumed that this omission was based on the fact that the surgery was described in the surgical note section of the discharge note. We chose this note because it enabled us to demonstrate several efficiencies in 1 short note. As shown in Figure 2C, 6 important information items from the original text, marked by yellow color, appeared in the H-summary but did not appear in the U-summary. In contrast, H-summary did not miss any items. Consequently, the calculated completeness for the H-summary was 100% (35/35), while the completeness of the U-summary was 84% (31/37 items of information), as shown also for note 15 in Table 2.

**Table 2:** Values of 2 metrics for 15 notes.

Notes	Word count of the original text^a^	Length reduction		Completeness	
		H- summaries^b^ (%)	U-summaries^c^ (%)	H-summaries, n/N (%)	U- summaries, n/N (%)
1	164	2	–10	35/35 (100)	29/35 (83)
2	366	23	20	31/32 (97)	28/32 (88)
3	160	1	21	40/40 (100)	39/40 (98)
4	266	23	26	41/42 (99)	41/42 (99)
5	377	5	7	24/26 (92)	22/26 (85)
6	448	39	31	32/33 (97)	28/33 (85)
7	386	25	21	43/46 (93)	38/46 (83)
8	312	24	27	21/22 (95)	20/22 (91)
9	180	–4	–6	22/23 (96)	22/23 (96)
10	560	33	44	33/35 (94)	27/35 (77)
11	520	36	39	24/27 (89)	23/27 (85)
12	375	38	37	38/42 (90)	35/42 (83)
13	411	40	34	42/43 (98)	41/43 (95)
14	155	33	19	29/30 (97)	27/30 (90)
15	128	13	29	37/37 (100)	31/37 (84)

^a^Mean 320, SD 139 words.

^b^H-summary: summary from highlighted notes (length reduction: mean 22, SD 15; completeness: mean 96, SD 4).

^c^U-summary: summary from unhighlighted notes (length reduction: mean 23, SD 15; completeness: mean 88, SD 6).

### Completeness

As shown in Table 2, on average, the completeness of the U-summaries was 88% (SD 6%), while the completeness of the H-summaries was 96% (SD 4%), which was 8% higher. All percentages in Table 2 are rounded to the nearest whole number. It is worth mentioning that for 13 notes, the completeness of the H-summary was higher than that of the U-summary, and for 2 notes, the completeness was equal. Using the Fisher exact test [52,53], we compared the number of notes with higher completeness in the H-summary group (13 notes) versus the number of notes with higher or equal completeness in the U-summary group (2 notes). The Fisher exact test yielded *P*=.01, indicating a statistically significant difference.

### Length Reduction

As shown in Table 2, the average word count of the original notes was 320 words, and the average length reduction of the H-summaries and U-summaries was 22% (SD 15%) and 23% (SD 15%) words, respectively. A negative number for length reduction in Table 2 indicates that the summary generated had more words than the original text.

### Correctness

In our analysis, we identified 3 instances of false information in U-summaries. In contrast, H-summaries contained only 2 false information items. However, this did not provide a statistically significant result, implying improvement. As an example of erroneous information, the original note contained the phrase “consistent either with aspiration pneumonia or increased apical interstitial edema,” whereas the U-summary incorrectly contained “consistent either with aspiration pneumonia and increased apical interstitial edema.” Replacing “or” with “and” changed the meaning of the phrase. Another example was a U-summary that mistakenly referred to Troponin T, a protein, as an enzyme.

### Structural Evaluation

The total number of SECs, which were either headers or bullet points, for the U-summaries was 516, while for H-summaries, it was 463. In terms of improper headers, there were 30 instances in the U-summaries compared to only 4 in the H-summaries. Table 3 presents the distribution of improper headers and misplaced information for the U-summaries and H-summaries for each of the 15 notes. For 11 (73%) of the notes, according to Table 3, the number of improper headers in the U-summaries was larger than the number for the H-summaries. For the other 4 (27%) notes, the numbers were equal. Hence, the number of improper headers in U-summaries was larger than those for H-summaries with statistical significance according to the Fisher exact test [52,53]. We compared the number of notes with a higher number of improper headers in the U-summary group (11 notes) and the number of notes with a higher number of improper headers in the H-summary group (4 notes). The Fisher exact test yielded *P*=.03, indicating a statistically significant result.

**Table 3:** Distribution of improper headers and misplaced information for the summaries from unhighlighted notes (U-summaries) and summaries from highlighted notes (H-summaries).

Notes	Improper headers		Misplaced information	
	U-summaries (n=30), n (%)	H-summaries, (n=4), n (%)	U-summaries, (n=8), n (%)	H-summaries, (n=2), n (%)
1	1 (3)	0 (0)	0 (0)	1 (50)
2	1 (3)	0 (0)	1 (12)	0 (0)
3	1 (3)	0 (0)	1 (12)	0 (0)
4	5 (17)	0 (0)	0 (0)	0 (0)
5	4 (13)	0 (0)	0 (0)	0 (0)
6	1 (3)	1 (25)	0 (0)	0 (0)
7	1 (3)	0 (0)	1 (12)	1 (50)
8	1 (3)	0 (0)	1 (12)	0 (0)
9	0 (0)	0 (0)	0 (0)	0 (0)
10	1 (3)	0 (0)	0 (0)	0 (0)
11	2 (7)	1 (25)	0 (0)	0 (0)
12	2 (7)	2 (50)	0 (0)	0 (0)
13	6 (20)	0 (0)	0 (0)	0 (0)
14	3 (10)	0 (0)	2 (25)	0 (0)
15	1 (3)	0 (0)	2 (25)	0 (0)

In other words, 5.8% (30/516) of the SECs in U-summaries were improper headers, whereas this percentage was only 0.1% (4/463) in the H-summaries. On the other hand, the number of proper SECs, after deducting the improper headers, was 94.2% (486/516) for the U-summaries and 99.1% (459/463) for the H-summaries. Hence, the structure of U-summaries was slightly more detailed than that of the H-summaries. However, the high percentage of improper headers rendered this advantage irrelevant because the structure was meaningfully less reliable. Moreover, we identified 8 instances of misplaced information in the U-summaries and only 2 in the H-summaries.

## Discussion

### Principal Findings

In this study, we evaluated summaries generated by LLMs from 2 versions of discharge notes, H-summaries and U-summaries, to test the hypothesis that highlighting discharge notes improves the accuracy of the generated summaries. Our results show that feeding LLMs with H-summaries, combined with prompt engineering, results in higher-quality summaries in terms of correctness, completeness, and structural integrity compared to U-summaries.

This study serves as the first step toward meeting the NIH challenge, which is facilitating the conversion of text in discharge notes into language that is comprehensible to a patient with a grade 6 reading level [6,60]. In a previous work [26], we conducted a study to simplify discharge notes using 2 simplification approaches.

In the first approach, we first summarized the discharge notes and then, in a second step, converted them into language understandable by a grade 6 reader. In the second approach, we directly generated, in a single step, a simplified summary understandable by a grade 6 reader from the discharge note. Our results showed that the first approach, simplifying notes in 2 steps, led to higher-quality and more understandable notes for grade 6 readers. Our interpretation is that, given discharge notes are inherently dense and complex, breaking the task into 2 steps reduces the cognitive load on the LLM, allowing it to generate more accurate and comprehensible results. Hence, cascading the findings of the previous study [26] to the findings of this paper provides a pipeline responding to the NIH challenge of summarizing discharge notes into a language that is comprehensible to a patient with a grade 6 reading level with high accuracy. Because the findings of both studies satisfy statistical significance, their combined impact is reliable, although this study is based on a sample of only 15 discharge notes.

Usually, summaries are shorter than the original text and thus enable the reader to review the content of a document faster. LLMs can actually generate summaries of various lengths according to the prompt provided to them. Because our final goal is the simplification of discharge notes, we are not necessarily interested in short summaries for the sake of brevity but in succinct summaries that capture all detailed information of a note in a simpler language.

When evaluating the summary of a discharge note, if the summary lacks key information from the original note (ie, low completeness), it cannot be considered accurate. Similarly, if the summary contains erroneous information (lacking correctness), its accuracy is also compromised. Furthermore, if relevant information is placed under incorrect categories (referred to as “misplaced information”), the summary’s accuracy is further affected. Therefore, in this paper, when we state that H-summaries have higher accuracy than U-summaries, we mean that H-summaries exhibit greater completeness, correctness, and structural integrity compared to U-summaries.

The scientific contribution of this research is the improvement in the accuracy of LLM summaries of discharge notes by feeding the LLMs with discharge notes in which the detailed information is highlighted. Automatic highlighting of discharge notes is provided by techniques from our previous research [28]. A sample of a discharge note highlighted automatically by this technique is shown in Figure 2A. Our hypothesis was that providing such highlighting would better direct the LLMs to capture the detailed information of the note and would better structure its summary. Our study has proven the plausibility of this hypothesis by the evaluation of several metrics.

Although highlighting is used in this paper, it is not the primary focus of our study. However, we wish to clarify an observation regarding the highlighting in Figure 2A. This example is rich in information but with some stop words not highlighted. While there are other notes in our sample where several words and phrases are not highlighted, we note that “discharge notes” from MIMIC-III EHRs have contributed to this richness of information as they summarize the hospital course of the patient. Our highlighting method is based on mining phrases from clinical notes and determining which ones should be incorporated into the interface terminology. Only concepts present in the interface terminology are subsequently highlighted within the clinical note. Indeed, the density of the highlighting depends on the type of clinical notes and will be higher in discharge notes due to the richness of information they contain. Full details can be found in the studies by Dehkordi et al [27,28].

Out of the 4 metrics we used to compare the H-summaries to the U-summaries, results from 2 metrics have shown statistical significance for better quality of the H-summaries. For the other 2 metrics, instances of erroneous information were low, but still, the results are better for H-summaries. The reason for having fewer improper headers in H-summaries compared to U-summaries may be attributed to the fact that by identifying the detailed information in the text, ChatGPT achieves better orientation in the note, allowing it to focus on the essential content and structure it more effectively. Despite the higher completeness in H-summaries compared to U-summaries, the inclusion of redundant information in U-summaries resulted in a similar length reduction for both H-summaries and U-summaries. For instance, the orange color in Figure 2B indicates that there is repetitive information in the U-summary, such as the phrase “one vessel coronary artery disease,” which appears twice.

We define SEC as the total number of headers and bullets. In general, a larger number of proper headers and bullets is desirable, as such categorization of information enhances the clarity of the notes, making them more understandable for the reader. Because this study represents the first step toward simplifying the texts, having information categorized in different sections to increase clarity is preferable, unless the headers are improper. Our study demonstrated that the ratio of improper headers is substantially lower in H-summaries compared to U-summaries.

Traditionally, for summarization, evaluation metrics such as recall-oriented understudy for gisting evaluation [61] are used. We have not used these metrics since (1) our hypothesis is comparing highlighted versus nonhighlighted notes with a focus on completeness, correctness, and structural integrity; and (2) there is a lack of human-generated summaries used by these metrics for computing the respective scores. Similarly, simplification of text is measured using metrics, such as the Flesch-Kincaid Grade Level [62], Simple Measure of Gobbledygook [63], and Gunning-Fog Index [64]. Because this study’s focus was not on the readability of the notes, these metrics were not applicable.

### Limitations

In our study, we considered only the discharge note part of the clinical notes, which are the most relevant parts of the clinical notes for the patients. Due to their summarizing nature, discharge notes have aspects from all other parts of clinical notes. Thus, we have chosen to concentrate only on the discharge note, while we expect a similar result for a study that would summarize the complete clinical note.

To minimize the amount of tedious manual review required for the evaluation of the various metrics, we have chosen a study of 15 notes, and this number was sufficient to yield the statistical significance for 2 dominant metrics of the 4 metrics for accuracy. For the other 2 metrics, the number of erroneous information items and misplaced information cases was infrequent, and a much larger sample would be needed to display statistical significance. Altogether, the results have proven our hypothesis that providing highlighted notes as input for ChatGPT will likely yield more accurate summaries.

Moreover, the reliance on extensive manual review for evaluating the summaries may introduce subjectivity into the assessment process. Future studies could benefit from developing more automated methods to assess summary quality to reduce potential bias and labor intensity. In addition, although highlighting is used to enhance the input for LLMs, the technique itself may have limitations, such as the potential for overhighlighting or missing critical information not recognized by the underlying algorithm. This could affect the quality of the input and, consequently, the accuracy of the summaries produced. However, we are continuously working in parallel on different automated methods to improve the highlighting techniques and algorithms [47,48]. Finally, this study’s results are based on the state of a specific LLM at a point in time. Continuous updates to LLMs could alter their performance characteristics, necessitating ongoing validation of the findings.

### Future Work

In future work, we will use the LLM H-summaries as a starting point for further simplification of the notes to meet NIH’s readability target for patients with grade 6 reading skills using various techniques. Some patients may be interested in just the big picture without many details, while others, based on their health literacy, may require detailed information. As mentioned previously, in the future, we intend to explore the capabilities of LLMs alongside the vast wealth of curated knowledge embedded in biomedical ontologies and terminologies in catering to the varied requirements of patient populations by offering summaries and simplifications of clinical notes tailored to individual needs and comprehension levels. While this study demonstrated statistical significance in the completeness and improper headers metrics, the limited sample size of 15 discharge notes was insufficient for the other 2 metrics. In future studies, we plan to use a larger sample to test statistical significance for the correctness and misplaced information metrics as well.

### Ethical Concerns

The integration of LLMs into clinical data summarization introduces several ethical considerations that warrant careful attention. A primary concern is the potential for these models to generate inaccurate or fabricated information, commonly referred to as hallucinations [65], which could adversely affect patient care.

Bias within training data presents another ethical challenge. If LLMs are trained on unrepresentative datasets, they may perpetuate existing health disparities by providing less accurate summaries for certain populations [66]. Addressing these biases is critical to ensure equitable health care delivery.

Moreover, the lack of transparency in how LLMs generate outputs [67] can lead to challenges in clinical settings. Clinicians may find it difficult to trust or validate AI-generated summaries without a clear understanding of the underlying processes, potentially hindering the integration of these tools into health care practice.

To mitigate these ethical concerns, it is imperative to implement robust validation processes, ensure compliance with privacy regulations, actively address biases in training data, and maintain transparency in AI operations.

### Conclusions

In this paper, we propose the use of highlighted discharge notes, emphasizing detailed information, to enhance the completeness and correctness of the summaries generated by LLMs. We have also developed and applied prompt engineering techniques to improve the structural integrity of these summaries, effectively addressing issues related to prompt sensitivity and reliability. Our study includes empirical validation with a random sample of 15 highlighted discharge notes from the MIMIC-III database, demonstrating that the input with the highlighted notes leads to more accurate LLM summaries of clinical notes. The final goal is to simplify discharge notes to be readable by a patient with grade 6 reading skills. To achieve this goal, we will use the summaries obtained in this research as input for the future simplification process.

## Abbreviations

AI: artificial intelligence
ATS: automatic text summarization
CIT: cardiology interface terminology
EHR: electronic health record
H-summary: summary from highlighted notes
HIPAA: Health Insurance Portability and Accountability Act
ICIT: initial version of cardiology interface terminology
LLM: large language model
NIH: National Institutes of Health
SEC: structural elements count
U-summary: summary from unhighlighted notes
